# Tumor-Infiltrating Immune Cells Act as a Marker for Prognosis in Colorectal Cancer

**DOI:** 10.3389/fimmu.2019.02368

**Published:** 2019-10-17

**Authors:** Lele Ye, Teming Zhang, Zhengchun Kang, Gangqiang Guo, Yongji Sun, Kangming Lin, Qunjia Huang, Xinyu Shi, Zhonglin Ni, Ning Ding, Kong-Nan Zhao, Wenjun Chang, Junjie Wang, Feng Lin, Xiangyang Xue

**Affiliations:** ^1^Department of Gynecologic Oncology, Wenzhou Central Hospital, Wenzhou, China; ^2^Department of Microbiology and Immunology, School of Basic Medical Sciences, Institute of Molecular Virology and Immunology, Institute of Tropical Medicine, Wenzhou Medical University, Wenzhou, China; ^3^Department of Gastrointestinal Surgery, The Second Affiliated Hospital, Wenzhou Medical University, Wenzhou, China; ^4^Department of Colorectal Surgery, Changhai Hospital, Naval Medical University, Shanghai, China; ^5^First Clinical College, Wenzhou Medical University, Wenzhou, China; ^6^Department of General Surgery, The Second Affiliated Hospital, Wenzhou Medical University, Wenzhou, China; ^7^Department of Environmental Health, Naval Medical University, Shanghai, China; ^8^Department of Critical Care Medicine, Shanghai Tenth People's Hosptial, Tongji University School of Medicine, Shanghai, China; ^9^Department of General Surgery, Taizhou First People's Hospital, Taizhou, China

**Keywords:** colorectal cancer, TIICs, TANs, Tregs, TAMs, prognosis, chemotherapy

## Abstract

Tumor-infiltrating immune cells (TIICs) play essential roles in cancer development and progression. However, the association of TIICs with prognosis in colorectal cancer (CRC) patients remains elusive. Infiltration of TIICs was assessed using ssGSEA and CIBERSORT tools. The association of TIICs with prognosis was analyzed in 1,802 CRC data downloaded from the GEO (https://www.ncbi.nlm.nih.gov/geo/) and TCGA (https://portal.gdc.cancer.gov/) databases. Three populations of TIICs, including CD66b+ tumor-associated neutrophils (TANs), FoxP3+ Tregs, and CD163+ tumor-associated macrophages (TAMs) were selected for immunohistochemistry (IHC) validation analysis in 1,008 CRC biopsies, and their influence on clinical features and prognosis of CRC patients was analyzed. Prognostic models were constructed based on the training cohort (359 patients). The models were further tested and verified in testing (249 patients) and validation cohorts (400 patients). Based on ssGSEA and CIBERSORT analysis, the correlation between TIICs and CRC prognosis was inconsistent in different datasets. Moreover, the results with disease-free survival (DFS) and overall survival (OS) data in the same dataset also differed. The high abundance of TIICs found by ssGSEA or CIBERSORT tools can be used for prognostic evaluation effectively. IHC results showed that TANs, Tregs, TAMs were significantly correlated with prognosis in CRC patients and were independent prognostic factors (*P*_DFS_ ≤ 0.001; *P*_OS_ ≤ 0.023). The prognostic predictive models were constructed based on the numbers of TANs, Tregs, TAMs (C-index_DFS&OS_ = 0.86; AIC_DFS_ = 448.43; AIC_OS_ = 184.30) and they were more reliable than traditional indicators for evaluating prognosis in CRC patients. Besides, TIICs may affect the response to chemotherapy. In conclusion, TIICs were correlated with clinical features and prognosis in patients with CRC and thus can be used as markers.

## Introduction

Colorectal cancer (CRC) is the second leading cause of cancer-related mortality (1). Some improvements have been made in diagnosis, chemoradiotherapy and surgery; however, morbidity and mortality of CRC remain high. The prognosis of patients with advanced tumors remains poorly understood (2). Currently, the prognosis of CRC patients mainly depends on the tumor status and TNM stage at diagnosis (3). However, none of these has independent prognostic value in CRC patients (4, 5). Therefore, it is necessary to develop predictive systems that can effectively predict the progression of the disease and the efficacy of chemotherapy in individual patients.

Accumulating evidence indicated that the features of tumor-infiltrating immune cells (TIICs) are correlated with the development and progression of cancer (6–8). The types and densities of TIICs not only have predictive value in patients' survival but also affect tumor responses to therapy, and therefore hold a great promise as clinical biomarkers for malignancies (9–13). Tumor-associated neutrophils (TANs), the predominant type of immune cells, eliminates pathogens and prevents the host from microbial infections (14) and positively correlate with worsened prognosis in breast cancer (15) and gastric cancer (16). Besides, namely tumor-associated macrophages (TAMs), can inhibit anti-tumor immunity, promote tumor progression and negatively correlated with the prognosis of CRC patients (17).

However, to the best of our knowledge, there are controversies in different experimental data regarding the correlation of TIICs with prognosis in CRC. TAN was positively correlated with worsened prognosis in CRC patients (18). However, Rao et al. reported the opposite conclusion (19). Several published reports have indicated that Tregs improved survival in CRC (20–22), while other authors have proposed Tregs as a risk factor for CRC (23, 24). Though TAMs were positively correlated with prognosis in 419 and 205 CRC patients (25, 26), Herrera and colleagues reported that TAMs were negatively correlated with prognosis in a retrospective study with 289 cases (27). Furthermore, some studies reported that infiltration of mast cells conferred survival advantage (28–30), whereas other reports indicated that it was associated with a worse prognosis (31–33). Recently, features of TIICs in tumor tissues have been analyzed by bioinformatics. Differences in the association of TIICs with prognosis were also found in different bioinformatics analyses (such as γδT) (34, 35).

These inconsistent results regarding the effect of TIIC infiltration on CRC and its correlation with patients' prognosis may result from many factors. Studies on only one or two types of TIICs with a small scale of samples (21, 23, 25, 36), or research that only used a single bioinformatics method without sufficient experimental validation (34, 37) are likely to yield unreliable results. Moreover, systematic studies analyzing the association of TIICs with the prognosis of CRC patients are lacking. Thus, a systematic study combining bioinformatics analysis and further verification based on a large scale of biopsies samples is required to characterize the association of TIICs with prognosis in patients with CRC.

**Figure 1:**
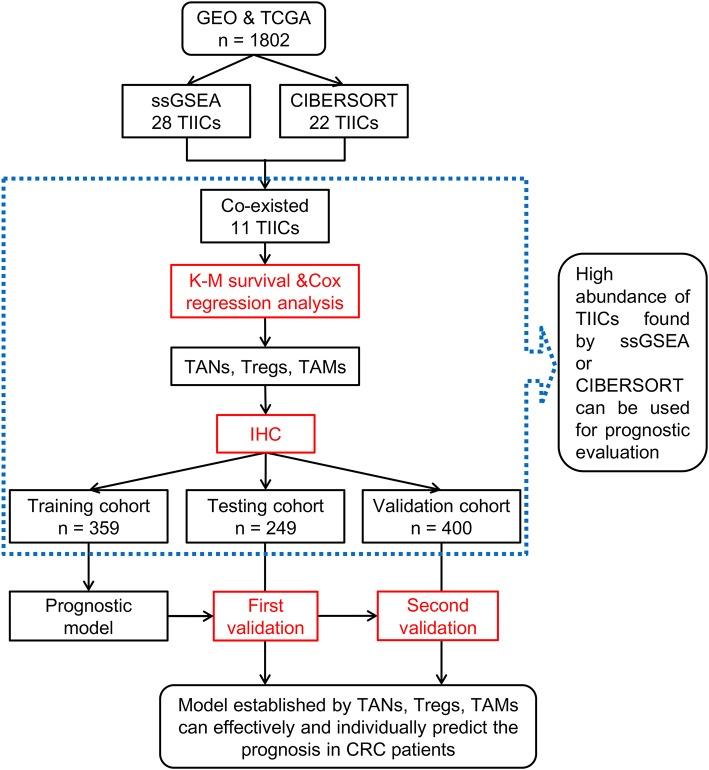
A flow chart of the research conducted.

In this study, we analyzed the association of CRC with TIICs using two machine-learning tools followed by immunohistochemistry (IHC) on a large scale of CRC biopsies. Moreover, we combined the chemotherapeutic data to further analyze the influence of TIICs on CRC. The flow chart of this study is shown in Figure 1.

## Materials and Methods

### Patients

Gene expression profiles (GEPs) from CRC patients' tissues were downloaded from GEO database (updated December 31, 2017; https://www.ncbi.nlm.nih.gov/geo/) using the following keywords: (“colorectal cancer” OR “colon cancer”) AND (“prognosis” OR “prognostic” OR “outcome” OR “survival” OR “progression” OR “recurrence” OR “relapse”) AND (“*Homo sapiens*”). The search retrieved 49 studies. We selected datasets sequenced using the GPL570 platform and excluded some datasets. The exclusion criteria were: (1) studies with <50 samples; (2) studies conducted using CRC cell lines; (3) studies without prognostic information; (4) studies with repeated datasets. The GSE14333, GSE17536, GSE17537, and GSE39582 datasets (1,107 CRC patients were included) were used in this study. In addition, these datasets contained disease-free survival (DFS) data for 949 patients and overall-survival (OS) data for 805 patients. GSE17536, GSE17537, and GSE39582 were merged into a single dataset as all of them contained both DFS and OS data. RNASeq data from 695 CRC patients and OS data for 469 patients among them were downloaded from TCGA database (https://portal.gdc.cancer.gov/; Supplementary Table 1).

For analysis of the clinical biopsies, 359 CRC samples from the Second Affiliated Hospital (Wenzhou Medical University; 2001–2009) and 649 CRC samples from the Changhai Hospital (Shanghai Naval Medical University; 2010–2011) were retrospectively collected. All the biopsies were consecutive samples acquired from surgical resection and processed immediately into formalin-fixed, paraffin-embedded tissues to produce the tissue array. Further, all samples were confirmed as CRC by two pathologists. The basic information, including rule-based post-operative chemotherapy (FOLFOX) and clinicopathological features of patients, were also collected (Supplementary Table 2). TNM stages were classified according to the American Joint Committee on Cancer guideline (7th Edition) but the detailed TNM categories (namely T, N, M) were lacking. It should be noted that there were two criteria for the category of patients according to their information related to lymph nodes (examined or metastatic) (38–40). Patients from the Second Affiliated Hospital (Wenzhou Medical University) were classified based on the number of examined lymph nodes during surgery. Patients from the Changhai Hospital (Shanghai Naval Medical University) were classified by the number of metastatic lymph nodes. Patients were divided into training (*n* = 359), testing (*n* = 249), and validation (*n* = 400) cohorts. This study was approved by the ethics committee of the Second Affiliated Hospital (Wenzhou Medical University) and Shanghai Changhai Hospital (Shanghai Naval Medical University). Informed consents were obtained from every CRC patient participating in this study.

### Analysis of Immune Cell Characteristics

GEPs data were analyzed using ssGSEA (41) and CIBERSORT (42). TIIC infiltration was classified as low or high abundance by using “maxstat” (R package).

#### ssGSEA

Normalized CRC GEPs data were compared with the gene set using “GSVA” (R package). ssGSEA classifies gene sets with common biological functions, chromosomal localization, and physiological regulation (41). The gene sets include 782 genes for predicting the abundance of 28 TIICs in individual tissue samples (http://software.broadinstitute.org/gsea/msigdb/index.jsp). The following 28 types of immune cells were obtained: activated B cells (Ba), activated CD4^+^ T cells (CD4^+^ Ta), activated CD8^+^ T cells (CD8^+^ Ta), activated dendritic cells (DCa), CD56^bright^ natural killer cells (CD56^+^ NK), CD56^dim^ natural killer cells (CD56^−^ NK), central memory CD4^+^ T cells (CD4^+^ Tcm), central memory CD8^+^ T cells (CD8^+^ Tcm), effector memory CD4^+^ T cells (CD4^+^ Tem), effector memory CD8^+^ T cells (CD8^+^ Tem), eosinophils, gamma delta T cells (γδT), immature B cells (Bi), immature dendritic cells (DCi), mast cells, myeloid-derived suppressor cells (MDSC), memory B cells (Bm), monocytes, natural killer cells (NK), natural killer T cells (NK T), neutrophils, plasmacytoid dendritic cells (DCp), macrophages, regulatory T cells (Tregs), follicular helper T cells (Tfh), type-1 T helper cells (Th1), type-17 T helper cells (Th17), and type-2 T helper cells (Th2). Normalized CRC GEP data were compared with the gene set to demonstrate the enrichment of 28 TIICs in CRC tissues (Supplementary Figure 1A).

#### CIBERSORT

The proportions of the 22 TIICs from each sample were determined by using the “CIBERSORT” (R package). CIBERSORT (42) was used to analyze the relative expression levels of 547 genes in individual tissue samples according to their GEPs, to predict the proportion of 22 types of TIICs in each tissue, namely: naive B cells (Bn), Bm, plasma cells, CD8^+^ T cells, naive CD4^+^ T cells (CD4^+^ Tn), CD4^+^ resting memory T cells (CD4^+^ Tmr), CD4^+^ memory-activated T cells (CD4^+^ Tma), Tfh, Tregs, γδT, resting natural killer cells (NKr), activated natural killer cells (NKa), monocytes, M0 macrophages (M0), M1 macrophages (M1), M2 macrophages (M2), resting dendritic cells (DCr), DCa, resting mast cells (Mr), activated mast cells (Ma), eosinophils, and neutrophils. Normalized CRC GEPs were transformed into the proportion of 22 TIICs. The relative expression of 22 TIICs in each sample was determined. Significant results (*P* < 0.05) were selected for subsequent analysis (Supplementary Figure 1B).

### Tissue Microarray (TMA) and IHC Experiments

Formalin-fixed, paraffin-embedded specimen arrays of consecutive CRC tissues from the training, testing, and validation cohorts were constructed as described previously (39). CD66b (43), FoxP3 (44), and CD163 (45) served as specific markers for tumor-associated neutrophils (TANs), regulatory T cells (Tregs), and tumor-associated macrophages (TAMs), respectively. After dewaxing in xylene, rehydrating in alcohol, and blocking endogenous peroxidase activity, TMAs were incubated overnight at 4°C with specific antibodies for CD66b (ab197678, rabbit; 1:100, Abcam, Cambridge, UK), FoxP3 (MAB8214; mouse; 1:200, BD Biosciences, USA), or CD163 (ab182422, rabbit; 1:500, Abcam). TMAs were then incubated at room temperature with secondary antibodies (ab97080, goat anti-rabbit, 1:2,000; ab97040, goat anti-mouse, 1:500, Abcam) for 10 min and 3-3′-diamino-benzidine for 1.5 min, then counterstained with hematoxylin for 30 s. The average number of positive cells was counted in three different fields of view in a 1 mm-diameter specimen. The numbers of TIICs were classified as low and high based on the median values.

### Statistical Analysis

Continuous variables were analyzed using Student's *t*-tests, *U*-tests, or nonparametric rank-sum tests. Categorical variables were analyzed using Chi-squared tests or Fisher's exact tests. Prognostic analyses were performed using Kaplan-Meier survival analysis and Cox univariate and multivariate analyses. Survival results were summarized using “forestplot” (R package). Prognostic predictive models were developed using the Cox regression coefficient and evaluated with the Akaike information criterion (AIC) and Harrell index of concordance (C-index). All data were analyzed by SPSS 22.0 for Windows (SPSS, Chicago, IL, USA) and R 3.5.0 (http://www.r-project.org/). The results with *P* < 0.05 were considered statistically significant.

## Results

### High Abundance of TIICs Found With ssGSEA or CIBERSORT Can be Used for Prognostic Analysis

**Figure 2:**
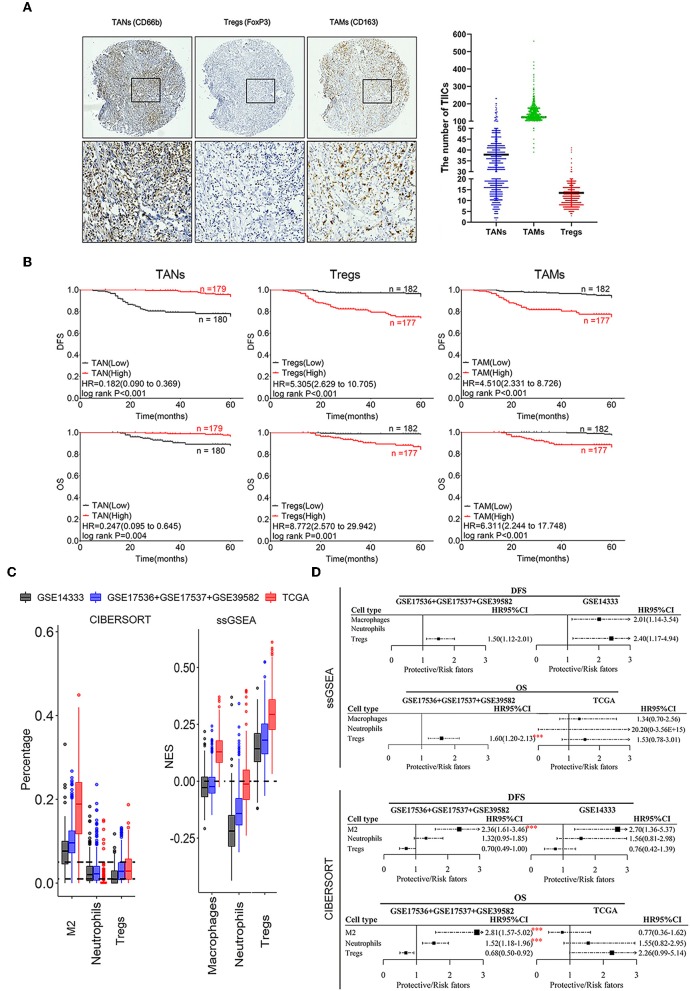
Correlation of infiltrating TANs, TAMs, and Tregs with prognosis in CRC patients. **(A)** The IHC images were taken at 100 (upper panel) and 400 (lower panel) magnification. **(B)** Association of the number of infiltrating TANs, Tregs, TAMs with CRC prognosis. **(C)** Infiltration of TANs, TAMs, and Tregs transformed by using ssGSEA and CIBERSORT. The relative numerical values corresponding to the height of the histogram indicate the different levels of abundance and the proportions. **(D)** Survival analysis: HR results from Cox univariate analysis with the transformed data based on ssGSEA and CIBERSORT tools. ***P ≤ 0.001.

There was an inconsistency in TIICs infiltration in patients from different datasets (such as Bm, DCa, macrophages, monocytes, γδT) based on both ssGSEA and CIBERSORT analysis (Supplementary Figure 1C). Moreover, no significant correlation was observed between the results of these two tools (Supplementary Figures 1D,E). Further, the associations between TIICs and CRC prognosis were also inconsistent in different datasets, such as Bm and γδT (Supplementary Figure 1F). Besides, different results using DFS and OS data were also obtained in the same dataset, such as γδT, M0, and Ma (Supplementary Figures 1F,G). The infiltration of four TIICs including macrophages M2, neutrophils, and Tregs was significantly negatively correlated with prognosis of CRC patients (*P* ≤ 0.001; Supplementary Figures 1F,G), and thus, these TIICs were chosen for IHC validation with 1,008 CRC tumor tissues (Figure 2A).

The prognostic analysis revealed that CRC patients with a high density of infiltrating TANs had a better prognosis, whereas those with a high number of infiltrating Tregs and TAMs had shorter DFS and OS (Figure 2B; Supplementary Figure 2). Cox univariate analysis showed that a high number of infiltrating TANs was an independent protective factor for DFS (*P* < 0.001) and OS (*P* ≤ 0.004; Supplementary Tables 3, 6, 9). The infiltration of Tregs and TAMs were independent risk factors for prognosis (*P* ≤ 0.001; Supplementary Tables 4, 5, 7, 8, 10, 11).

The infiltration of neutrophils showed a relatively lower abundance, compared to macrophages M2 and Tregs that showed relatively high enrichment in CRC tissues by using ssGSEA and CIBERSORT tools (Figure 2C). Moreover, high infiltration of these three populations was related to the short survival of CRC patients (*P* ≤ 0.001; Figures 2C,D). Combined with the above results, we suggested that the prognostic evaluation based on the high abundance of TIICs assessed through ssGSEA or CIBERSORT analysis is reliable.

### Association of TANs, Tregs, and TAMs With Several Clinical Features in CRC Patients

**Figure 3:**
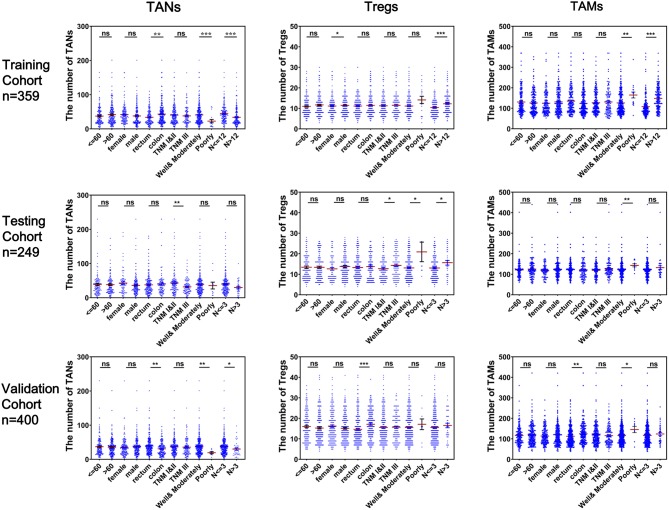
Correlation of infiltrating TANs, Tregs, and TAMs with clinical features in CRC patients. Each dot represents one patient.

As shown in Figure 3, CRC tissues from patients with well-to-moderate tumor differentiation, fewer numbers of lymph nodes examined or metastases, TNM stage I or II disease or rectum cancer generally tended to have higher TAN abundances and lower Treg or TAM abundances in the three cohorts (*P* < 0.05). Associations between the numbers of these three TIICs and other clinical features were not statistically significant. The results based on infiltrating levels of TAN, Treg, and TAM were consistent with those from continuous variables in the training cohort (Table 1).

**Table 1:** Correlation between TANs, Tregs, and TAMs and clinical features in the training cohort (*n* = 359).

	**TANs**			**Tregs**			**TAMs**		
	**Low**	**High**	***P*-value^**#**^**	**Low**	**High**	***P*-value^**#**^**	**Low**	**High**	***P*-value^**#**^**
**Characteristics**	**(*n* = 180)**	**(*n* = 179)**		**(*n* = 182)**	**(*n* = 177)**		**(*n* = 182)**	**(*n* = 177)**	
Age [*n* (%)]			0.369			0.125			0.877
≤60	93 (51.7%)	84 (46.9%)		97 (53.3%)	80 (45.2%)		89 (48.9%)	88 (49.7%)	
>60	87 (48.3%)	95 (53.1%)		85 (46.7%)	97 (54.8%)		93 (51.1%)	89 (50.3%)	
Gender [*n* (%)]			**0.002**			0.843			0.14
Women	63 (35.0%)	91 (50.8%)		79 (43.4%)	75 (42.4%)		85 (46.7%)	69 (39.0%)	
Men	117 (65.0%)	88 (49.2%)		103 (56.6%)	102 (57.6%)		97 (53.3%)	108 (61.0%)	
Disease location [*n* (%)]			**0.035**			0.281			0.196
Rectum	82 (45.6%)	62 (34.6%)		68 (37.4%)	76 (42.9%)		67 (36.8%)	77 (43.5%)	
Colon	98 (54.4%)	117 (65.4%)		114 (62.6%)	101 (57.1%)		115 (63.2%)	100 (56.5%)	
TNM stage [*n* (%)]			0.190			0.682			0.968
I and II	115 (63.9%)	126 (70.4%)		124 (68.1%)	117 (66.1%)		122 (67.0%)	119 (67.2%)	
III	65 (36.1%)	53 (29.6%)		58 (31.9%)	60 (33.9%)		60 (33.0%)	58 (32.8%)	
Differentiation [*n* (%)]			**0.037**			0.301			0.001^*^
Well and moderately	155 (86.1%)	168 (93.9%)		168 (92.3%)	155 (87.6%)		172 (94.5%)	151 (85.3%)	
Poorly	13 (7.2%)	4 (2.2%)		6 (3.3%)	11 (6.2%)		1 (0.5%)	16 (9.0%)	
Missing	12 (6.7%)	7 (3.9%)		8 (4.4%)	11 (6.2%)		9 (4.9%)	10 (5.6%)	
Number of lymph nodes (examined) [*n* (%)]			0.001^*^			**0.001**			0.001^*^
≤12	66 (36.7%)	113 (63.1%)		106 (58.2%)	73 (41.2%)		123 (67.6%)	56 (31.6%)	
>12	114 (63.3%)	66 (36.9%)		76 (41.8%)	104 (58.8%)		59 (32.4%)	121 (68.4%)	
Serum CEA [*n* (%)]			0.863			0.830			0.389
<5	107 (59.4%)	108 (60.3%)		108 (59.3%)	107 (60.5%)		105 (57.7%)	110 (62.1%)	
≥5	73 (40.6%)	71 (39.7%)		74 (40.7%)	70 (39.5%)		77 (42.3%)	67 (37.9%)	
Serum CA199 [*n* (%)]			0.909			0.170			0.591
<37	148 (82.2%)	148 (82.7%)		155 (85.2%)	141 (79.7%)		152 (83.5%)	144 (81.4%)	
≥37	32 (17.8%)	31 (17.3%)		27 (14.8%)	36 (20.3%)		30 (16.5%)	33 (18.6%)	

# *χ^2^-test or Fisher's exact test. TNM, tumor to node to metastasis; CEA, carcinoembryonic antigen; CA, carbohydrate antigen*.

* *The P values < 0.05 were bold*.

### Construction and Evaluation of Prognostic Models

Efficient prognostic predictive models were constructed based on the infiltrating number of TANs, Tregs, and TAMs from the training cohort. Compared with the models from traditional TNM-staging evaluation, our models were more reliable and effective for determining the prognosis of patients with CRC (C-indexe_DFS&OS_: 0.86; AIC_DFS_: 448.43, AIC_OS_: 184.30). Surprisingly, the C-index and AIC of these two models were approached those of the models constructed by combing TAN, Treg, TAM infiltration with the TNM stage (Table 2). The risk-calculation formulas for prognosis (DFS & OS) in patients with CRC were as follows:

(1) $$\begin{array}{l} Risk\left(DFS\right)=0.1605\;Tregs+0.0078\;TAMs-0.0593\;TANs \end{array}$$

(2) $$\begin{array}{l} Risk\left(OS\right)=0.1692\;Tregs+0.0046\;TAMs-0.0374\;TANs \end{array}$$

**Figure 4:**
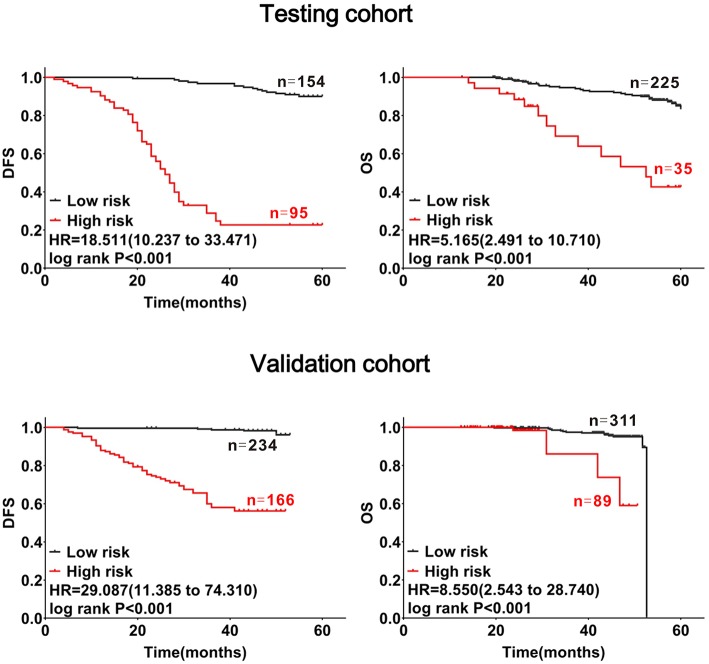
Confirmation of the prognostic value of the mathematical model with CRC patients. Patients with CRC were divided into high- and low-risk subgroups at cut-off points of 1.44705 for DFS and 3.4513 for OS. The red lines represent high-risk groups of patients with CRC with substantial decreases in DFS and OS, the black lines represent low-risk groups of patients with CRC with relatively stable DFS and OS.

Using these formulas, CRC patients were divided into low- and high-risk groups based on cut-off values obtained from receiver-operating curves (Supplementary Figure 3). As shown in the testing and validation cohorts, patients with CRC in the high-risk group had a poorer prognosis (log-rank *P*_*DFS&OS*_ < 0.001, Figure 4).

**Table 2:** Comparison of the prognostic accuracies of different models developed by the training cohort.

**DFS**			**OS**		
**Composition of model**	**C-index**	**AIC**	**Composition of model**	**C-index**	**AIC**
TNM	0.61	537.59	TNM	0.56	237.11
TANs + Tregs + TAMs	0.86	448.43	TANs + Tregs + TAMs	0.86	184.30
TNM + TANs + Tregs + TAMs	0.87	439.70	TNM + TANs + Tregs + TAMs	0.86	186.00

*AIC, Akaike information criterion; C-index, Harrell index of concordance*.

### Association of TANs, Tregs, and TAMs With Chemotherapeutic Efficiency in CRC Patients

**Figure 5:**
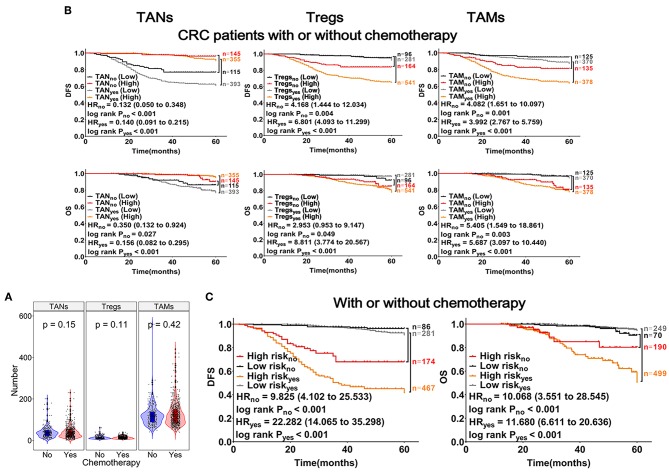
Effects of TANs, Tregs, and TAMs on chemotherapeutic efficacy in patients with CRC. **(A)** Patients with CRC were divided into chemotherapy (*n* = 748) and non-chemotherapy (*n* = 260) groups. Each dot represents one patient, patients treated with or without chemotherapy represented with red or blue columns, respectively. **(B)** Results from Kaplan-Meier and Cox regression analyses performed with TANs, Tregs, and TAMs shown within the graphs. **(C)** Risk analysis of the effects of chemotherapy on prognosis in patients with CRC, using the mathematical model described in Figure 4.

The abundances (Infiltrating number) of TANs, Tregs, and TAMs were not significantly different between the chemotherapy and non-chemotherapy groups (Figure 5A). CRC patients with a higher abundance of TANs or lower abundance of Tregs and TAMs, had a longer DFS and OS, which were independent of chemotherapy (Figure 5B). Moreover, CRC patients with high-risk scores in the above predictive models also had a shorter DFS and OS (log-rank *P*_*DFS&OS*_ < 0.001; Figure 5C). Interestingly, among CRC patients who had received chemotherapy, differences in prognosis between patients with varying abundances of TANs, Tregs, and TAMs were more remarkable. Besides, these differences were more prominent for DFS (log-rank *P*_*DFS&OS*_ < 0.001, Figures 5B,C).

## Discussion

In this study, we systematically analyzed the role of TIICs in CRC using bioinformatics tools and IHC validation on a large scale of samples. Several studies have reported the infiltration features of TIICs in CRC tissues using only one bioinformatics tool, and have analyzed the association of TIICs with prognosis with or without validation in a small scale of samples (34, 35, 46, 47). Unlike previous studies, we combined two bioinformatics analysis tools, i.e., ssGSEA and CIBERSORT, to systematically analyze the association of TIIC abundances with CRC samples data obtained from TCGA and GEO databases, respectively. We found that the results of 11 co-existing TIICs observed by these two tools were different in the same dataset. Moreover, the abundances of TIICs and their associations with CRC prognosis analyzed using the same tool were also inconsistent in different datasets. Based on the bioinformatics analysis, TANs, TAMs, and Tregs were significantly correlated with worse prognosis in patients with CRC. Thus, these three types of TIICs were chosen for IHC detection in 1,008 CRC samples to further validate their roles in CRC development and progression. The trend of TAMs infiltration detected by IHC was consistent with that obtained from ssGSEA and CIBERSORT analyses in this study. Surprisingly, the infiltration of TANs detected by IHC was inconsistent with that detected by these two bioinformatics analyses. Similar observations were found in Tregs using ssGSEA. Recent studies have revealed the heterogeneity of immune-related genes in different ethnic groups (48, 49). These inconsistent results may be due to the use of different datasets with variations in ethnic groups, sex, age, and sample quality used for detecting GEPs. In addition, the use of different markers for cell population may also affect survival analysis. Therefore, a large number of markers for detecting TIICs are urgently required to reduce the variation in results in the future. Nevertheless, by combing the results from online public databases analysis and validation by IHC, we conclude that the high abundance of TIICs found by ssGSEA or CIBERSORT analysis can be used for prognostic analysis. Besides, we suggested that ssGSEA and CIBERSORT need to be further optimized.

Similar to previous reports (27, 36, 50), our data further confirmed that the infiltration of TAMs and Tregs were independent prognostic risk factors in CRC and that TAN infiltration was an independent protective prognostic factor. Some studies have reported that TANs, Tregs, and TAMs are mainly found in poorly differentiated or undifferentiated and late-TNM stage of CRC (26, 36), and another study have reported that TANs and Tregs mainly infiltrated tumors with low or moderately differentiation (21). In this study, the relationships between the clinical features of patients with CRC and the abundances of TANs, Tregs, and TAMs showed similar trends in three verified cohorts, although there were several mixed findings. These inconsistent results may be due to the fact that tumor immunity is dynamic during tumor development and progression. The dynamic changes in TIICs in terms of the immune cell type, quantity, and proportion, as well as the differences in sampling time and location, may have contributed to the observed heterogeneities among the different populations studied.

The traditional prognostic evaluation of patients with CRC mainly relies on the TNM stage. While the TNM stage is valuable to predict prognosis, it is limited by its lack of information at the cellular and molecular levels, which may contribute to the considerable heterogeneities in terms of clinical outcomes of patients with CRC, even with identical TNM stages. In this context, there have been numerous studies searching for a single biomarker as a better prognostic indicator for CRC as well as other tumors, and integrating multiple elements into a model could substantially improve the prognostic value over that of the single biomarker in many cancers (51–53). Here, we established a novel prognostic model by combining the numbers of TANs, Tregs, and TAMs. This could overcome the prognostic limitations in these patients with the same TNM stage. In addition, this prognostic model could be effectively used to assess the risk score of an individual patient, meaning that the models can be used as markers for prognosis of CRC patients.

Immune cells can mediate chemotherapeutic resistance and sensitivity to improve survival in patients with CRC (54, 55). Unfortunately, some datasets from the online public databases did not contain information about patients' chemotherapy. The sample size could also affect results, and thus, correlations analysis between TIICs and chemotherapy were not conducted for the samples from the databases. Here, we found that CRC patients with lower number of TAMs or Tregs, higher number of TANs had longer survival, regardless of chemotherapy. However, differences in prognosis between the groups with different number of TIICs were more distinct in the patients with chemotherapy, similar to the observations with the prognostic model. These findings were further supported in our analysis of DFS. In this way, we supposed that CRC patients with a low-risk score based on the prognostic models may benefit from chemotherapy. However, further study is needed to explore and define the interactions of TIICs with chemotherapy.

Although this study has made progress compared with previous studies, there are still some limitations in this study. Firstly, only three types of immune cells were studied by IHC validation in this study, while other cell populations were lacking. In addition, since the samples studied in this study were all collected from 2001 to 2011, some clinical and molecular-level variables such as (K)RAS, BRAF, MSI/MSH, which have an important impact on modern prognosis and therapy, were not available and covered in this study, which may result in bias to some extent. Therefore, a prospective study with comprehensive coverage of cell populations in tumor immune microenvironment, including sufficient clinical information related to modern precision therapy, is required to systematically explore the role of TIICs in CRC genesis and development.

In conclusion, this study systematically evaluated the landscape of immune cells in CRC tissue samples with two bioinformatics tools. We found that TANs, Tregs, and TAMs were all independent prognostic factors in both databases and collected samples. Novel prognostic predictive models were constructed from the number of infiltrating TANs, Tregs, and TAMs. These models could facilitate the evaluation of prognosis and identification of patients with CRC who are more suitable for chemotherapy. These findings reveal the significant role of TIICs in CRC and could have implications for postoperative personalized follow-up, care, action, management, and decision-making regarding individualized chemotherapy for CRC.

## Data Availability Statement

The datasets generated during and/or analyzed during the current study are available from the corresponding author on reasonable request. Besides, the accession number(s) and direct links to these datasets are as follow: GSE14333: https://www.ncbi.nlm.nih.gov/geo/query/acc.cgi?acc=GSE14333; GSE17536: https://www.ncbi.nlm.nih.gov/geo/query/acc.cgi?acc=GSE17536; GSE17537: https://www.ncbi.nlm.nih.gov/geo/query/acc.cgi?acc=GSE17537; GSE39582: https://www.ncbi.nlm.nih.gov/geo/query/acc.cgi?acc=GSE39582; TCGA: https://portal.gdc.cancer.gov/repository.

## Ethics Statement

The studies involving human participants were reviewed and approved by Ethics committee of the Second Affiliated Hospital (Wenzhou Medical University, China) and Shanghai Changhai Hospital (Shanghai Naval Medical University, China). The patients/participants provided their written informed consent to participate in this study. Written informed consent was obtained from the individual(s) for the publication of any potentially identifiable images or data included in this article.

## Author Contributions

LY performed the experiments, analyzed and interpreted the data, and drafted the manuscript. TZ interpreted the data and contributed to the substantial revisions of the manuscript. ZN and ZK performed the experiments. GG, TZ, and K-NZ helped to perform the statistical analysis and interpret the data. YS, KL, QH, XS, TZ, ND, and WC contributed to sample collection, acquisition of patients' clinical and survival data. FL and JW acquired the material support and coordinated the study. XX made contribution to the conception and design, analyzed and interpreted the data, supervised the study, provided the project funding, revised the manuscript, and finally approved the version of the manuscript for publication. All authors read and approved the final manuscript.

### Conflict of Interest

The authors declare that the research was conducted in the absence of any commercial or financial relationships that could be construed as a potential conflict of interest.

## Abbreviations

CD4+ Tma: CD4 positive activated memory T cell
CD4+ Tmr: CD4 positive resting memory T cell
CD8+ T cell: CD8 positive T cell
CRC: colorectal cancer
DCa: activated dendritic cell
DFS: disease-free survival
GEPs: gene expression profiles
IHC: immunohistochemistry
NK: natural killer
OS: overall survival
TAM: tumor-associated macrophage
TAN: tumor-associated neutrophil
Tfh: T follicular helper cell
TMA: tissue microarray
Treg: regulatory T cell
TIIC: tumor-infiltrating immune cell.
