# Supplementary material for: Tumor-Infiltrating Immune Cells Act as a Marker for Prognosis in Colorectal Cancer
Source: Front Immunol. 2019 Oct 17;10:2368. doi: 10.3389/fimmu.2019.02368 (PMC6811516; doi:10.3389/fimmu.2019.02368)
Supplement: Supplementary file 1 [file Data_Sheet_1.docx]

Supplementary Material

# Supplementary Figures and Tables

## Supplementary Figures

**
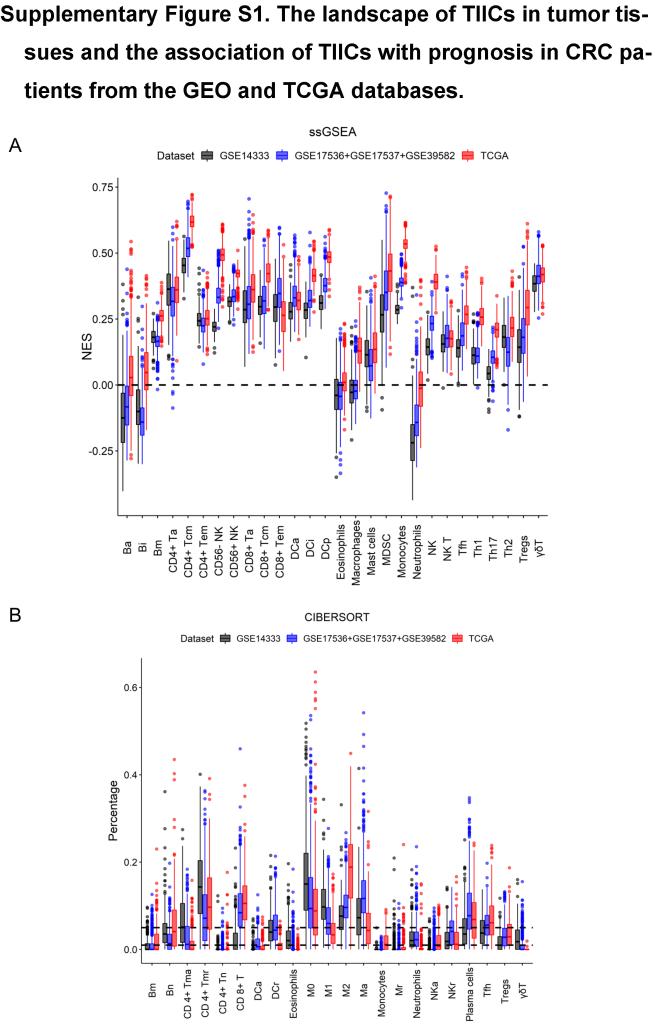

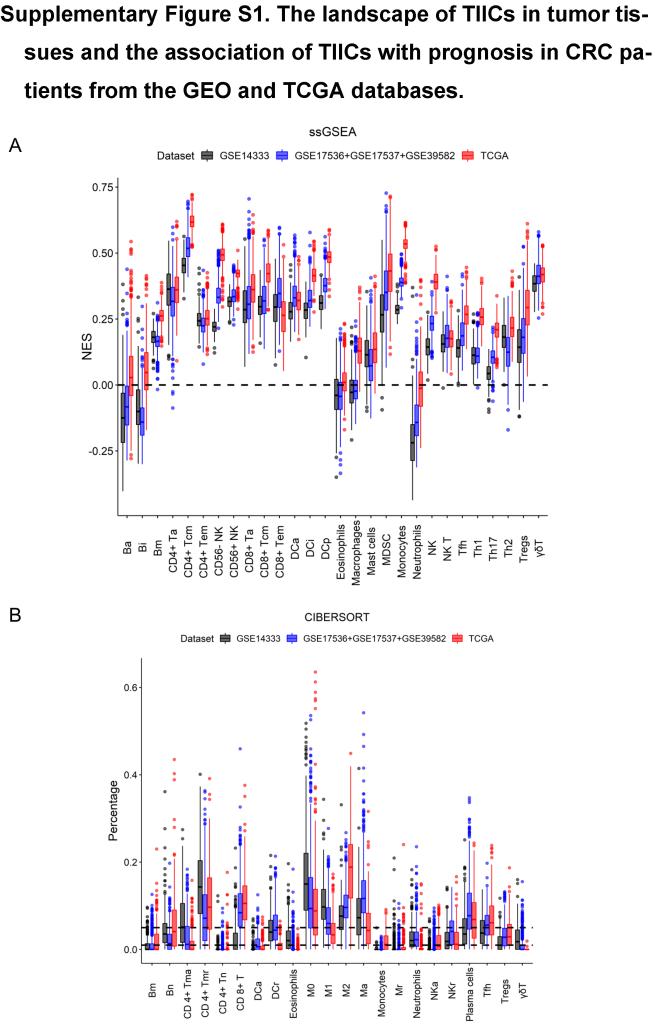
**
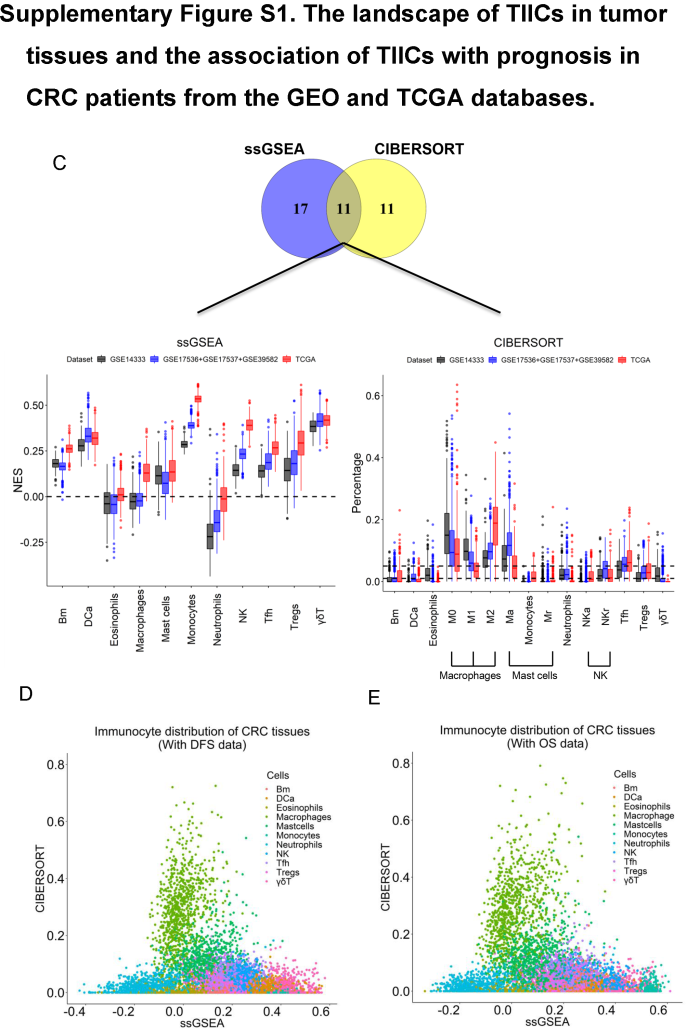


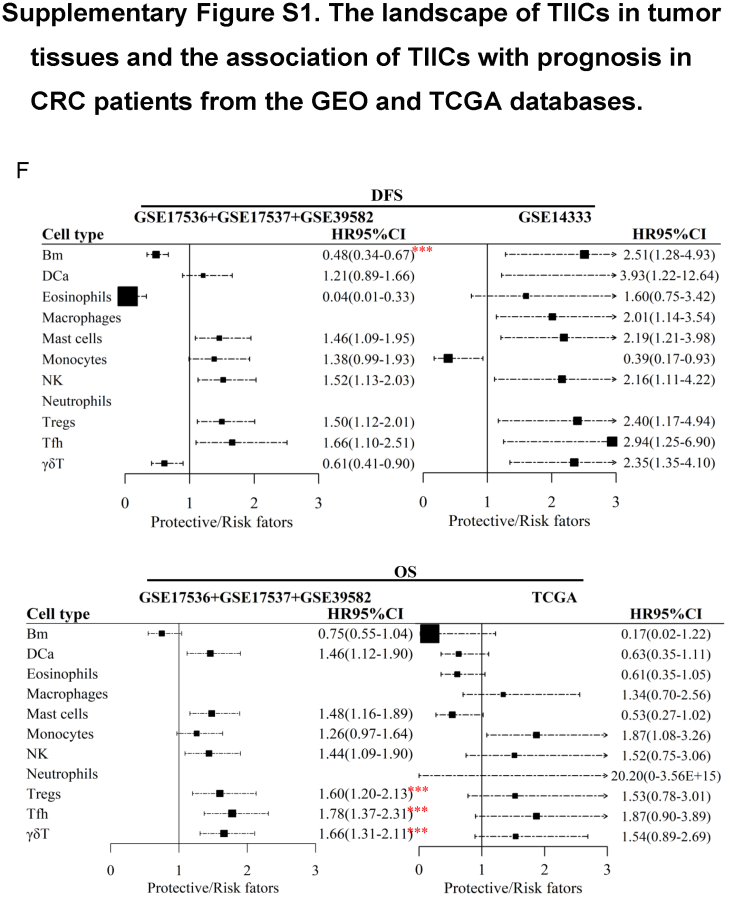

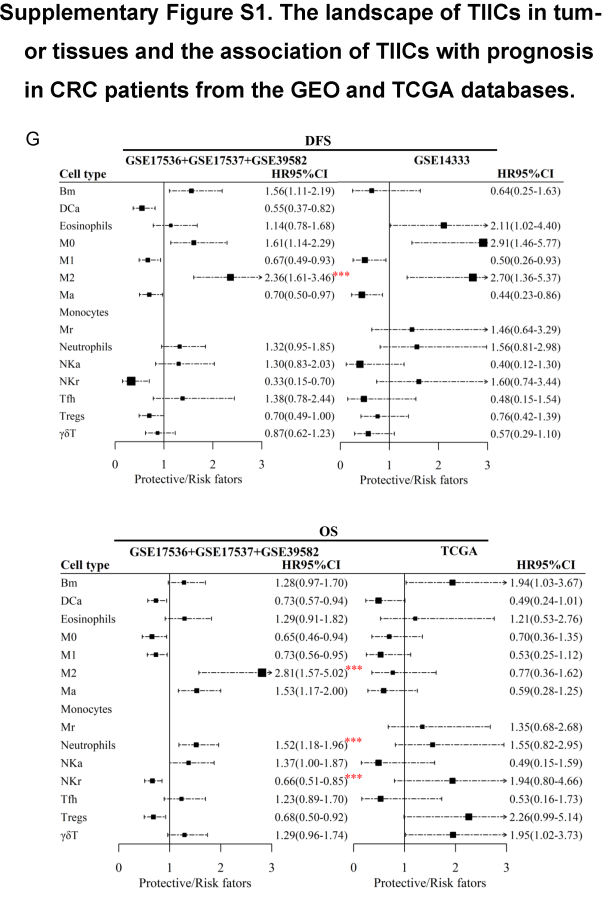


### Supplementary Figure 1. The landscape of TIICs in tumor tissues and the association of TIICs with prognosis in CRC patients from the GEO and TCGA databases.

**(A)** Results of all TIICs transformed using ssGSEA. The NES values of 28 TIICs in three datasets were analyzed using ssGSEA. Twenty-three TIICs were positively abundant (NES > 0), and only neutrophils were negatively abundant (NES < 0). The numerical values corresponding to the height of the histogram indicate different abundance levels, where < 0 indicated that the degree of enrichment was poor, and a larger value indicated greater enrichment. **(B)** The percentages of 22 TIICs were analyzed using CIBERSORT. The numerical values corresponding to the height of the histogram indicate differing proportions. Larger values indicated higher ratios. Four cell types (CD4^+^ Tmrs, macrophages M0, macrophages M2, and activated mast cells) accounted for more than 5% of all cells and were consistent in different datasets. Naive B cells, plasma, CD8^+^ T cells, CD4^+^ Tmas, Tfh cells, Tregs, NK resting cells, macrophages M1, and neutrophils accounted for more than 1% of all cells. Other cell types accounted for less than or equal to 1% of all cells, as shown in the graph. **(C)** Among the identified TIICs, we re-identified macrophages (M0, M1, and M2), NK cells (activated and resting), and mast cells (activated and resting). Eleven TIIC types were found in common with both tools. Among them, ssGSEA analysis revealed that activated dendritic cells (DCas), monocytes, natural killer (NK) cells, gamma delta T cells (γδT) and Tregs were enriched. CIBERSORT analysis showed that macrophages and mast cells were relatively enriched in CRC tissue; **(D)** Correlation of the abundance of 11 TIICs identified using ssGSEA with their proportion estimated by CIBERSORT in 813 patients with CRC, according to DFS data. Each dot represented one patient, and each color represented one of the 11 types of TIICs; **(E)** Correlation of the abundance of 11 TIICs identified by ssGSEA and CIBERSORT in 853 patients with CRC, based on OS data; **(F)** Based on the ssGSEA tools, Cox regression analysis showed that the infiltration of DCas, macrophages, mast cells, NK cells, Tregs, and Tfh cells were negatively correlated with prognosis in ssGSEA, and only eosinophils and monocytes were positively correlated; **(G)** CIBERSORT analysis showed that infiltration of DCas, macrophages M1, and NK resting cells (NKrs) were positively correlated with prognosis, whereas infiltration of memory B cells, eosinophils, macrophages M2, and neutrophils were negatively correlated. ****P* ≤ 0.001.

**
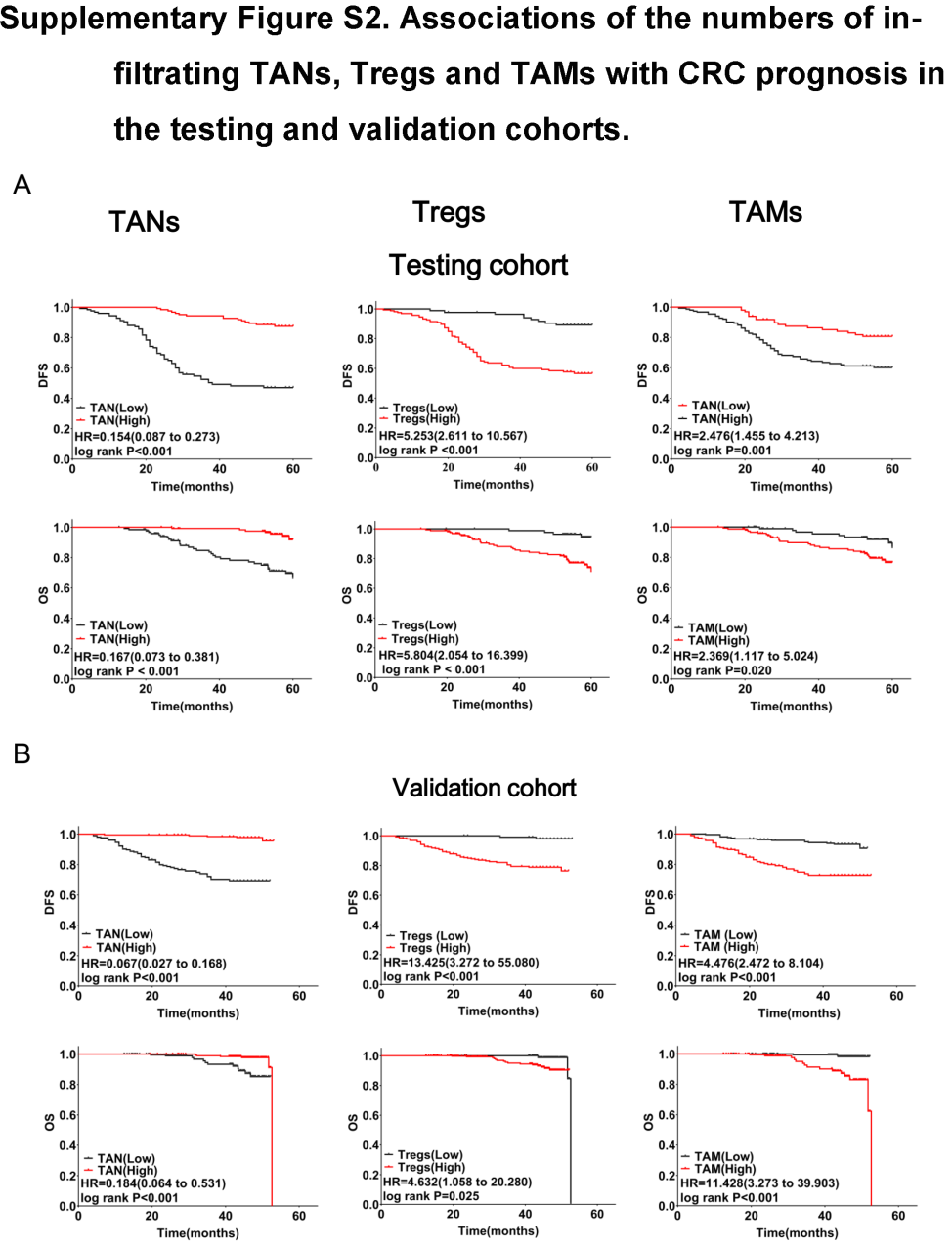
**

### Supplementary Figure 2. Associations of the number of infiltrating TANs, Tregs and TAMs with CRC prognosis in the testing and validation cohorts.

**(A)** Results of prognostic values of TANs, Tregs, and TAMs were analyzed and compared by Kaplan-Meier survival and Cox regression analyses, which were carried out using SPSS for the testing **(A)** and validation cohorts **(B)**. The red line indicated a group with a higher number of cells, and the black line indicated a group with a lower number of cells.

**
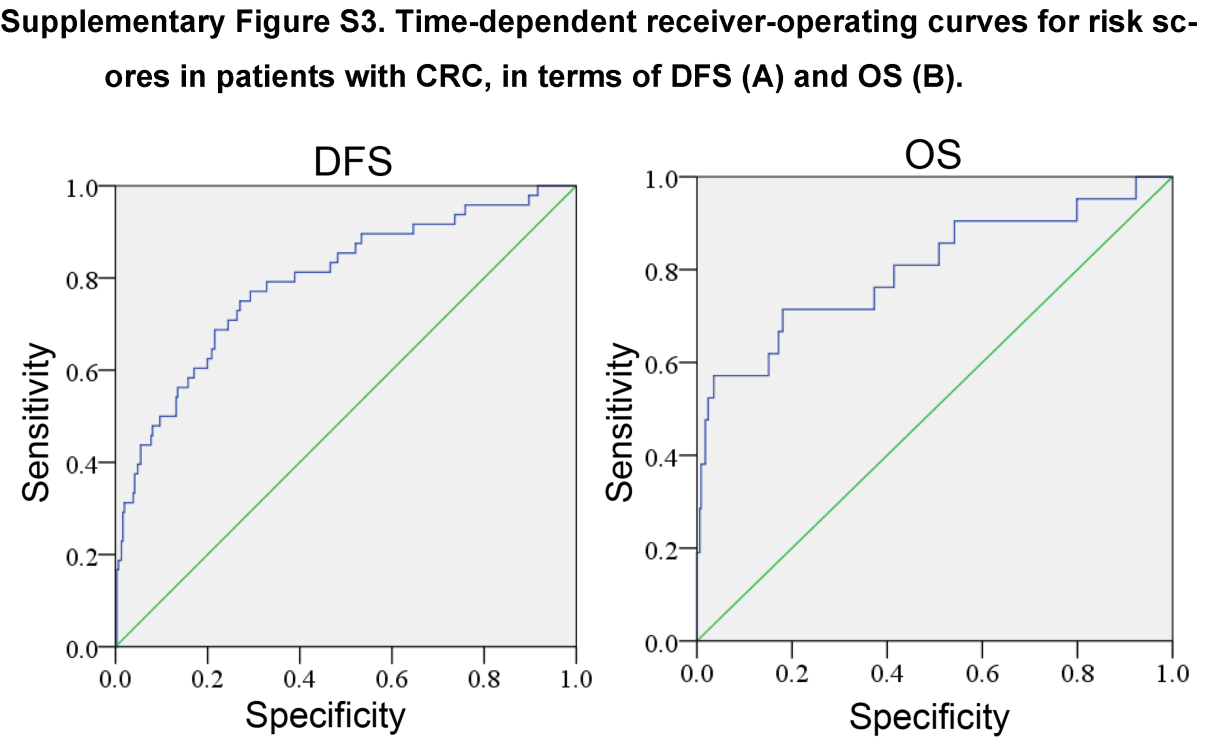
**

### Supplementary Figure 3. Time-dependent receiver-operating curves for risk scores in patients with CRC, in terms of DFS (A) and OS (B).

## Supplementary Tables

### Supplementary Table 1. Population demographics from the GEO and TCGA databases.

| Supplementary Table 1. Population demographics from the GEO and TCGA databases. | | | | | | | | |
| --- | --- | --- | --- | --- | --- | --- | --- | --- |
| Study cohorts | Platform | Samples | ssGSEA | DFS | OS | CIBERSORT(*P* < 0.05) | DFS | OS |
| GSE14333 | Affymetrix (GPL570) | 290 | 290 | 226 | / | 241 | 192 | / |
| GSE17536 + GSE17537 + GSE39582 | Affymetrix (GPL570) | 817 | 817 | 723 | / | 705 | 621 | / |
| GSE17536 + GSE17537 + GSE39582 | Affymetrix (GPL570) | 817 | 817 | / | 805 | 705 | / | 694 |
| TCGA | Illumina | 695 | 695 | / | 469 | 206 | / | 199 |
| * Same dataset with different DFS and OS data. | |  |  |  |  |  |  |  |

### Supplementary Table 2. Distribution of the IHC population in this study.

| Supplementary Table 2. Distribution of the IHC population in this study. | |  |  |
| --- | --- | --- | --- |
| Characteristics | Training cohort (n = 359, Wenzhou) | Testing cohort (n = 249, Shanghai) | Validation cohort (n = 400, Shanghai) |
| Age (n (%)) |  |  |  |
| ≤ 60 | 177 (49.3%) | 131 (52.6%) | 184 (46.0%) |
| ＞ 60 | 182 (50.7%) | 118 (47.4%) | 216 (54.0%) |
| Gender (n (%)) |  |  |  |
| Women | 154 (42.9%) | 84 (33.7%) | 174 (43.5%) |
| Men | 205 (57.1%) | 165 (66.3%) | 226 (56.5%) |
| Disease location (n (%)) |  |  |  |
| Rectum | 144 (40.1%) | 165 (66.3%) | 244 (61.0%) |
| Colon | 215 (59.9%) | 84 (33.7%) | 156 (39.0%) |
| TNM stage (n (%)) |  |  |  |
| Ⅰ & Ⅱ | 241 (67.1%) | 143 (57.4%) | 270 (67.5%) |
| Ⅲ | 118 (32.9%) | 106 (42.6%) | 130 (32.5%) |
| Differentiation (n (%)) |  |  |  |
| Well & Moderately | 323 (90.0%) | 239 (96.0%) | 391 (97.8%) |
| Poorly | 17 ( 4.7%) | 10 ( 4.0%) | 9 ( 2.2%) |
| Missing | 19 (5.3%) | 0 (0%) | 0 (0%) |
| Number of lymph nodes (examined or metastatic) (n (%))* | |  |  |
| ≤ 12 or ≤ 3 | 179 (49.9%) | 213 (85.5%) | 357 (89.3%) |
| ＞ 12 or > 3 | 180 (50.1%) | 36 (14.5%) | 40 (10.0%) |
| Missing | 0 (0%) | 0 (0%) | 3 (0.8%) |
| Chemotherapy (yes or no) (n (%)) |  |  |  |
| Yes | 303 (84.40%) | 180 (72.29%) | 265 (66.25%) |
| No | 56 (15.60%) | 69 (27.71%) | 135 (33.75%) |
| CEA (n (%)) |  |  |  |
| ＜ 5 | 215 (59.9%) | 166 (66.7%) | 252 (63.0%) |
| ≥ 5 | 144 (40.1%) | 82 (32.9%) | 142 (35.5%) |
| Missing | 0 (0%) | 1 (0.40%) | 6 (1.5%) |
| CA199 (n (%)) |  |  |  |
| ＜ 37 | 296 (82.5%) | 221 (88.8%) | 341 (85.25%) |
| ≥ 37 | 63 (17.5%) | 27 (10.8%) | 52 (13.0%) |
| Missing | 0 (0%) | 1 (0.4%) | 7 (1.75%) |
| Abbreviations: TNM, tumour node metastasis; CEA, carcinoembryonic antigen; CA, carbohydrate antigen. * 12 was the cut-off value in examed (Training cohort), 3 was the cut-off value in metastasis (Testing and Validation cohorts). | | | |

### Supplementary Table 3. Cox regression analysis of TANs and clinicopathological covariates with disease-free survival and overall survival in the training cohort.

| Supplementary Table 3. Cox regression analysis of TANs and clinicopathological covariates with disease-free survival and overall survival in the training cohort. | | | | | | | | | | |  | |
| --- | --- | --- | --- | --- | --- | --- | --- | --- | --- | --- | --- | --- |
|  | Disease-free survival | | | | |  | Overall survival | | | | |  |
|  | Univariate analysis | |  | Multivariate analysis | |  | Univariate analysis | |  | Multivariate analysis | | |
| Variables | HR (95% CI) | P-value |  | HR (95% CI) | P-value |  | HR (95% CI) | P-value |  | HR (95% CI) | P-value | |
| TANs (Low vs High) | 0.182 (0.090 to 0.369) | **0.001*** |  | 0.239 (0.115 to 0.496) | **0.001*** |  | 0.247 (0.095 to 0.645) | **0.004** |  | 0.305 (0.114 to 0.815) | **0.018** | |
| Age (≤ 60 vs ＞ 60 years) | 1.136 (0.644 to 2.005) | 0.659 |  |  |  |  | 1.569 (0.650 to 3.786) | 0.316 |  |  |  | |
| Gender (female vs male) | 1.609 (0.883 to 2.934) | 0.120 |  |  |  |  | 1.113 (0.469 to 2.643) | 0.808 |  |  |  | |
| Disease location (rectum vs colon) | 1.382 (0.750 to 2.546) | 0.299 |  |  |  |  | 1.222 (0.493 to 3.029) | 0.666 |  |  |  | |
| TNM stage (I&II vs III) | 2.524 (1.430 to 4.455) | **0.001** |  | 3.320 (1.796 to 6.136) | **0.001*** |  | 1.571 (0.661 to 3.729) | 0.306 |  |  |  | |
| Differentiation grade (well & moderately vs poorly) | 8.206 (4.8048 to 16.633) | **0.001*** |  | 9.432 (4.415 to 20.151) | **0.001*** |  | 10.602 (3.822 to 29.412) | **0.001*** |  | 8.239 (2.929 to 23.172) | **0.001*** | |
| Chemotherapy (yes vs No) | 2.911 (0.904 to 9.366) | 0.073 |  |  |  |  | 1.834 (0.427 to 7.874) | 0.415 |  |  |  | |
| Number of lymph nodes examined (≤ 3 vs ＞ 3) | 2.074 (1.471 to 4.971) | **0.001** |  | 2.273 (1.212 to 4.262) | **0.010** |  | 1.669 (0.700 to 3.980) | 0.248 |  |  |  | |
| Serum CEA (ng/ml) (< 5 vs ≥ 5) | 1.317 (0.746 to 2.323) | 0.342 |  |  |  |  | 2.052 (0.865 to 4.870) | 0.103 |  |  |  | |
| Serum CA19-9 (U/ml) (< 37 vs ≥ 37) | 1.344 (0.670 to 2.699) | 0.405 |  |  |  |  | 1.153 (0.388 to 3.429) | 0.798 |  |  |  | |
| Abbreviations: HR = hazard ratio; TNM, tumour to node to metastasis; CEA, carcinoembryonic antigen; CA, carbohydrate antigen. *: P value < 0.001 | | | | | | | | | | | |  |
|  | | | | | | | | | | | |  |

### Supplementary Table 4. Cox regression analysis of Tregs and clinicopathological covariates with disease-free survival and overall survival in the training cohort.

| Supplementary Table 4. Cox regression analysis of Tregs and clinicopathological covariates with disease-free survival and overall survival in the training cohort. | | | | | | | | | | | |  |
| --- | --- | --- | --- | --- | --- | --- | --- | --- | --- | --- | --- | --- |
|  | Disease-free survival | | | | |  | Overall survival | | | | | |
|  | Univariate analysis | |  | Multivariate analysis | |  | Univariate analysis | |  | Multivariate analysis | | |
| Variables | HR (95% CI) | P-value |  | HR (95% CI) | P-value |  | HR (95% CI) | P-value |  | HR (95% CI) | P-value | |
| Tregs (Low vs High) | 5.305 (2.629 to 10.705) | **0.001*** |  | 4.340 (2.121 to 8.878) | **0.001*** |  | 8.772 (2.570 to 29.942) | **0.001** |  | 7.230 (2.096 to 24.937) | **0.002** | |
| Age (≤ 60 vs ＞ 60 years) | 1.136 (0.644 to 2.005) | 0.659 |  |  |  |  | 1.569 (0.650 to 3.786) | 0.316 |  |  |  | |
| Gender (female vs male) | 1.609 (0.883 to 2.934) | 0.12 |  |  |  |  | 1.113 (0.469 to 2.643) | 0.808 |  |  |  | |
| Disease location (rectum vs colon) | 1.382 (0.750 to 2.546) | 0.299 |  |  |  |  | 1.222 (0.493 to 3.029) | 0.666 |  |  |  | |
| TNM stage (I&II vs III) | 2.524 (1.430 to 4.455) | **0.001** |  | 3.466 (1.882 to 6.381) | **0.001*** |  | 1.571 (0.661 to 3.729) | 0.306 |  |  |  | |
| Differentiation grade (well&moderately vs poorly) | 8.206 (4.8048 to 16.633) | **0.001*** |  | 9.065 (4.263 to 19.277) | **0.001*** |  | 10.602 (3.822 to 29.412) | **0.001*** |  | 7.783 (2.801 to 21.620) | **0.001*** | |
| Chemotherapy (yes vs No) | 2.911 (0.904 to 9.366) | 0.073 |  |  |  |  | 1.834 (0.427 to 7.874) | 0.415 |  |  |  | |
| Number of lymph nodes examined (≤ 3 vs ＞ 3) | 2.074 (1.471 to 4.971) | **0.001** |  | 2.591 (1.380 to 4.864) | **0.003** |  | 1.669 (0.700 to 3.980) | 0.248 |  |  |  | |
| Serum CEA (ng/ml) (< 5 vs ≥ 5) | 1.317 (0.746 to 2.323) | 0.342 |  |  |  |  | 2.052 (0.865 to 4.870) | 0.103 |  |  |  | |
| Serum CA19-9 (U/ml) (< 37 vs ≥ 37) | 1.344 (0.670 to 2.699) | 0.405 |  |  |  |  | 1.153 (0.388 to 3.429) | 0.798 |  |  |  | |
| Abbreviations: HR = hazard ratio; TNM, tumour to node to metastasis; CEA, carcinoembryonic antigen; CA, carbohydrate antigen. *: P value < 0.001 | | | | | | | | | | | | |
| Supplementary Table 5. Cox regression analysis of TAMs abundances versus clinicopathological covariates with disease-free survival and overall survival in the training cohort. Supplementary Table 5. Cox regression analysis of TAMs abundances versus clinicopathological covariates with disease-free survival and overall survival in the training cohort. | | | | | | | | | | | |  |
|  | Disease-free survival | | | | |  | Overall survival | | | | | |
|  | Univariate analysis | |  | Multivariate analysis | |  | Univariate analysis | |  | Multivariate analysis | | |
| Variables | HR (95% CI) | P-value |  | HR (95% CI) | P-value |  | HR (95% CI) | P-value |  | HR (95% CI) | | P-value |
| TAMs (Low vs High) | 4.510 (2.331 to 8.726) | **0.001*** |  | 1.013 (1.008 to 1.018) | **0.001*** |  | 6.311 (2.244 to 17.748) | **0.001*** |  | 4.336 (1.454 to 12.932) | | **0.009** |
| Age (≤ 60 vs ＞ 60 years) | 1.136 (0.644 to 2.005) | 0.659 |  |  |  |  | 1.569 (0.650 to 3.786) | 0.316 |  |  | |  |
| Gender (female vs male) | 1.609 (0.883 to 2.934) | 0.12 |  |  |  |  | 1.113 (0.469 to 2.643) | 0.808 |  |  | |  |
| Disease location (rectum vs colon) | 1.382 (0.750 to 2.546) | 0.299 |  |  |  |  | 1.222 (0.493 to 3.029) | 0.666 |  |  | |  |
| TNM stage (I&II vs III) | 2.524 (1.430 to 4.455) | **0.001** |  | 3.492 (1.863 to 6.546) | **0.001*** |  | 1.571 (0.661 to 3.729) | 0.306 |  |  | |  |
| Differentiation grade (well&moderately vs poorly) | 8.206 (4.8048 to 16.633) | **0.001*** |  | 8.519 (3.934 to 18.447) | **0.001*** |  | 10.602 (3.822 to 29.412) | **0.001*** |  | 5.440 (1.865 to 15.868) | | **0.002** |
| Chemotherapy (yes vs No) | 2.911 (0.904 to 9.366) | 0.073 |  |  |  |  | 1.834 (0.427 to 7.874) | 0.415 |  |  | |  |
| Number of lymph nodes examined (≤ 3 vs ＞ 3) | 2.074 (1.471 to 4.971) | **0.001** |  | 1.924 (1.021 to 3.627) | **0.043** |  | 1.669 (0.700 to 3.980) | 0.248 |  |  | |  |
| Serum CEA (ng/ml) (< 5 vs ≥ 5) | 1.317 (0.746 to 2.323) | 0.342 |  |  |  |  | 2.052 (0.865 to 4.870) | 0.103 |  |  | |  |
| Serum CA19-9 (U/ml) (< 37 vs ≥ 37) | 1.344 (0.670 to 2.699) | 0.405 |  |  |  |  | 1.153 (0.388 to 3.429) | 0.798 |  |  | |  |
| Abbreviations: HR = hazard ratio; TNM, tumor to node to metastasis; CEA, carcinoembryonic antigen; CA, carbohydrate antigen. *: P value < 0.001 | | | | | |  |  |  |  |  | |  |

### Supplementary Table 6. Cox regression analysis of TAN abundances versus clinicopathological covariates with disease-free survival and overall survival in the testing cohort.

| Supplementary Table 6. Cox regression analysis of TAN abundances versus clinicopathological covariates with disease-free survival and overall survival in the testing cohort. | | | | | | | | | | | |
| --- | --- | --- | --- | --- | --- | --- | --- | --- | --- | --- | --- |
|  | Disease-free survival | | | | |  | Overall survival | | | | |
|  | Univariate analysis | |  | Multivariate analysis | |  | Univariate analysis | |  | Multivariate analysis | |
| Variables | HR (95% CI) | P-value |  | HR (95% CI) | P-value |  | HR (95% CI) | P-value |  | HR (95% CI) | P-value |
| TANs (Low vs High) | 0.154 (0.087 to 0.273) | **0.001*** |  | 0.160 (0.090 to 0.283) | **0.001*** |  | 0.167 (0.073 to 0.381) | **0.001*** |  | 0.174 (0.076 to 0.396) | **0.001*** |
| Age (≤ 60 vs > 60 years) | 0.634 (0.395 to 1.118) | 0.059 |  |  |  |  | 0.969 (0.508 to 1.850) | 0.924 |  |  |  |
| Gender (female vs male) | 1.063 (0.654 to 1.730) | 0.805 |  |  |  |  | 1.402 (0.678 to 2.895) | 0.361 |  |  |  |
| Disease location (rectum vs colon) | 0.957 (0.588 to 1.558) | 0.861 |  |  |  |  | 0.795 (0.393 to 1.610) | 0.525 |  |  |  |
| TNM stage (I&II vs III) | 2.505 (1.570 to 3.999) | **0.001*** |  |  |  |  | 2.509 (1.290 to 4.877) | **0.007** |  |  |  |
| Differentition grade (well&moderately vs poorly) | 1.604 (0.586 to 4.395) | 0.358 |  |  |  |  | 1.470 (0.353 to 6.111) | 0.596 |  |  |  |
| Chemotherapy(yes vs no) | 2.392 (1.260 to 4.540) | **0.008** |  |  |  |  | 1.454 (0.664 to 3.182) | 0.349 |  |  |  |
| Number of metastatic lymph nodes (≤ 3 vs > 3) | 2.574 (1.527 to 4.339) | **0.001*** |  | 2.164 (1.274 to 3.675) | **0.004** |  | 3.164 (1.561 to 6.415) | **0.001** |  | 2.885 (1.420 to 5.862) | **0.003** |
| Serum CEA (ng/ml) (< 5 vs ≥ 5) | 2.025 (1.279 to 3.204) | **0.003** |  |  |  |  | 2.025 (1.059 to 3.871) | **0.033** |  |  |  |
| Serum CA19-9 (U/ml) (< 37 vs ≥ 37) | 2.930 (1.658 to 5.179) | **0.001*** |  | 2.202 (1.233 to 3.934) | **0.008** |  | 2.744 (1.253 to 6.008) | **0.012** |  |  |  |
| Abbreviations: HR = hazard ratio; TNM, tumor to node to metastasis; CEA, carcinoembryonic antigen; CA19-9, carbohydrate antigen. *: P value < 0.001 | | | | | | | | | | | |

### Supplementary Table 7. Cox regression analysis of Treg abundances versus clinicopathological covariates with disease-free survival and overall survival in the testing cohort.

| Supplementary Table 7. Cox regression analysis of Treg abundances versus clinicopathological covariates with disease-free survival and overall survival in the testing cohort. | | | | | | | | | | | | | | |  |
| --- | --- | --- | --- | --- | --- | --- | --- | --- | --- | --- | --- | --- | --- | --- | --- |
|  | Disease-free survival | | | | |  | Overall survival | | | | | | | | |
|  | Univariate analysis | |  | Multivariate analysis | |  | Univariate analysis | | | |  | Multivariate analysis | | | |
| Variables | HR (95% CI) | P-value |  | HR (95% CI) | P-value |  | | HR (95% CI) | P-value |  | | | HR (95% CI) | P-value |  |
| Tregs (Low vs High) | 5.253(2.611 to 10.567) | **0.001*** |  | 4.883 (2.422 to 9.841) | **0.001*** |  | | 5.804 (2.054 to 16.399) | **0.001** |  | | | 5.658 (2.001 to 15.996) | **0.001** |  |
| Age (≤ 60 vs > 60 years) | 0.634 (0.395 to 1.118) | 0.059 |  |  |  |  | | 0.969 (0.508 to 1.850) | 0.924 |  | | |  |  |  |
| Gender (female vs male) | 1.063 (0.654 to 1.730) | 0.805 |  |  |  |  | | 1.401 (0.678 to 2.895) | 0.362 |  | | |  |  |  |
| Disease location (rectum vs colon) | 0.957 (0.588 to 1.558) | 0.861 |  |  |  |  | | 0.795 (0.393 to 1.610) | 0.525 |  | | |  |  |  |
| TNM stage (I&II vs III) | 2.505 (1.570 to 3.999) | **0.001*** |  | 2.073 (1.281 to 3.355) | **0.003** |  | | 2.509 (1.290 to 4.877) | **0.007** |  | | |  |  |  |
| Differentiation grade (well&moderately vs poorly) | 1.604 (0.586 to 4.395) | 0.358 |  |  |  |  | | 1.470 (0.353 to 6.111) | 0.596 |  | | |  |  |  |
| Chemotherapy (yes vs no) | 2.392 (1.260 to 4.540) | **0.008** |  |  |  |  | | 1.454 (0.664 to 3.182) | 0.349 |  | | |  |  |  |
| Number of metastatic lymph nodes (≤ 3 vs > 3) | 2.574 (1.527 to 4.339) | **0.001*** |  |  |  |  | | 3.164 (1.561 to 6.415) | **0.001** |  | | | 3.015 (1.485 to 6.121) | **0.002** |  |
| Serum CEA (ng/ml) (< 5 vs ≥ 5) | 2.025 (1.279 to 3.204) | **0.003** |  | 2.135 (1.188 to 3.838) | **0.001*** |  | | 2.025 (1.059 to 3.871) | **0.033** |  | | |  |  |  |
| Serum CA19-9 (U/ml) (<37 vs ≥37) | 2.930 (1.658 to 5.179) | **0.001*** |  |  |  |  | | 2.744 (1.253 to 6.008) | **0.012** |  | | |  |  |  |
| Abbreviations: HR = hazard ratio; TNM, tumor to node to metastasis; CEA, carcinoembryonic antigen; CA, carbohydrate antigen. *: P value < 0.001. | | | | | | | | | | | | | | |  |

### Supplementary Table 8. Cox regression analysis of TAM abundances versus clinicopathological covariates with disease-free survival and overall survival in the testing cohort.

| Supplementary Table 8. Cox regression analysis of TAM abundances versus clinicopathological covariates with disease-free survival and overall survival in the testing cohort. | | | | | | | | |  |  |  |
| --- | --- | --- | --- | --- | --- | --- | --- | --- | --- | --- | --- |
|  | Disease-free survival | | | | |  | Overall survival | | | | |
|  | Univariate analysis | |  | Multivariate analysis | |  | Univariate analysis | |  | Multivariate analysis | |
| Variables | HR (95% CI) | P-value |  | HR (95% CI) | P-value |  | HR (95% CI) | P-value |  | HR (95% CI) | P-value |
| TAMs (Low vs High) | 2.476 (1.455 to 4.213) | **0.001** |  | 2.753 (1.614 to 4.696) | **0.001*** |  | 2.369 (1.117 to 5.024) | **0.025** |  | 2.568 (1.207 to 5.464) | **0.014** |
| Age (≤ 60 vs > 60 years) | 0.634 (0.395 to 1.118) | 0.059 |  |  |  |  | 0.969 (0.508 to 1.850) | 0.924 |  |  |  |
| Gender (female vs male) | 1.063 (0.654 to 1.730) | 0.805 |  |  |  |  | 1.401 (0.678 to 2.895) | 0.362 |  |  |  |
| Disease location (rectum vs colon) | 0.957 (0.588 to 1.558) | 0.861 |  |  |  |  | 0.795 (0.393 to 1.610) | 0.525 |  |  |  |
| TNM stage (I&II vs III) | 2.505 (1.570 to 3.999) | **0.001*** |  | 2.517 (1.571 to 4.034) | **0.001*** |  | 2.509 (1.290 to 4.877) | **0.007** |  |  |  |
| Differentiation grade (well&moderately vs poorly) | 1.604 (0.586 to 4.395) | 0.358 |  |  |  |  | 1.470 (0.353 to 6.111) | 0.596 |  |  |  |
| Chemotherapy (yes vs no) | 2.392 (1.260 to 4.540) | **0.008** |  |  |  |  | 1.454 (0.664 to 3.182) | 0.349 |  |  |  |
| Number of metastatic lymph nodes (≤ 3 vs > 3) | 2.574 (1.527 to 4.339) | **0.001*** |  |  |  |  | 3.164 (1.561 to 6.415) | **0.001** |  | 3.445 (1.693 to 7.010) | **0.001*** |
| Serum CEA (ng/ml) (< 5 vs ≥ 5) | 2.025 (1.279 to 3.204) | **0.003** |  | 1.925 (1.213 to 3.055) | **0.005** |  | 2.025 (1.059 to 3.871) | **0.033** |  |  |  |
| Serum CA19-9 (U/ml) (< 37 vs ≥ 37) | 2.930 (1.658 to 5.179) | **0.001*** |  |  |  |  | 2.744 (1.253 to 6.008) | **0.012** |  |  |  |
| Abbreviations: HR = hazard ratio; TNM, tumor to node to metastasis; CEA, carcinoembryonic antigen; CA, carbohydrate antigen. *: P value < 0.001. | | | | | | | | | | | |

### Supplementary Table 9. Cox regression analysis of TAN abundances versus clinicopathological covariates with disease-free survival and overall survival in the validation cohort.

| Supplementary Table 9. Cox regression analysis of TAN abundances versus clinicopathological covariates with disease-free survival and overall survival in the validation cohort. | | | | | | | | | | | |
| --- | --- | --- | --- | --- | --- | --- | --- | --- | --- | --- | --- |
|  | Disease-free survival | | | | |  | Overall survival | | | | |
|  | Univariate analysis | |  | Multivariate analysis | |  | Univariate analysis | |  | Multivariate analysis | |
| Variables | HR (95% CI) | P-value |  | HR (95% CI) | P-value |  | HR (95% CI) | P-value |  | HR (95% CI) | P-value |
| TANs (Low vs High) | 0.067 (0.027 to 0.168) | **0.001*** |  | 0.067 (0.027 to 0.168） | **0.001*** |  | 0.184 (0.064 to 0.531) | **0.002** |  | 0.165 (0.054 to 0.507) | **0.002** |
| Age (≤ 60 vs > 60 years) | 1.310 (0.774 to 2.218) | 0.314 |  |  |  |  | 0.457 (0.169 to 1.235) | 0.123 |  |  |  |
| Gender (female vs male) | 0.564 (0.335 to 0.949) | **0.031** |  |  |  |  | 0.507 (0.193 to 1.334) | 0.169 |  |  |  |
| Disease location (rectum vs colon) | 1.708 (1.017 to 2.869) | **0.043** |  |  |  |  | 4.623 (1.704 to 12.543) | **0.003** |  | 5.715 (2.065 to 15.817) | **0.001** |
| TNM stage (I&II vs III) | 1.461 (0.863 to 2.474) | 0.158 |  |  |  |  | 0.309 (0.071 to 1.350) | 0.119 |  |  |  |
| Differentition grade (well&moderately vs poorly) | 4.390 (1.585 to 12.158) | **0.004** |  |  |  |  | 11.092 (2.512 to 48.985) | **0.001** |  | 4.468 (0.970 to 20.584) | **0.055** |
| Chemotherapy (yes vs no) | 1.657 (0.908 to 3.026) | 0.100 |  |  |  |  | 0.986 (0.363 to 2.678) | 0.978 |  |  |  |
| Number of metastatic lymph nodes (≤ 3 vs > 3) | 2.138 (1.080 to 4.234) | **0.029** |  |  |  |  | 0.705 (0.093 to 5.337) | 0.735 |  |  |  |
| Serum CEA (ng/ml) (< 5 vs ≥ 5) | 1.447 (0.860 to 2.435) | 0.164 |  |  |  |  | 1.257 (0.456 to 3.463) | 0.658 |  |  |  |
| Serum CA19-9 (U/ml) (< 37 vs ≥ 37) | 1.667 (0.864 to 3.215) | 0.127 |  |  |  |  | 1.110 (0.253 to 4.862) | 0.890 |  |  |  |
| Abbreviations: HR = hazard ratio; TNM, tumor to node to metastasis; CEA, carcinoembryonic antigen; CA, carbohydrate antigen. *: P value < 0.001. | | | | | | | | | | | |

### Supplementary Table 10. Cox regression analysis of Treg abundances versus clinicopathological covariates with disease-free survival and overall survival in the validation cohort.

| Supplementary Table 10. Cox regression analysis of Treg abundances versus clinicopathological covariates with disease-free survival and overall survival in the validation cohort. | | | | | | | | | | | |
| --- | --- | --- | --- | --- | --- | --- | --- | --- | --- | --- | --- |
|  | Disease-free survival | | | | |  | Overall survival | | | | |
|  | Univariate analysis | |  | Multivariate analysis | |  | Univariate analysis | |  | Multivariate analysis | |
| Variables | HR (95% CI) | P-value |  | HR (95% CI) | P-value |  | HR (95% CI) | P-value |  | HR (95% CI) | P-value |
| Tregs (Low vs High) | 13.425 (3.272 to 55.080) | **0.001*** |  | 13.669 (3.326 to 56.177) | **0.001*** |  | 4.632 (1.058 to 20.280) | **0.042** |  | 5.940 (1.282 to 27.531) | **0.023** |
| Age (≤60 vs >60 years) | 1.310 (0.774 to 2.218) | 0.314 |  |  |  |  | 0.457 (0.169 to 1.235) | 0.123 |  |  |  |
| Gender (female vs male) | 0.564 (0.335 to 0.949) | **0.031** |  | 0.543 (0.321 to 0.920) | **0.023** |  | 0.507 (0.193 to 1.334) | 0.169 |  |  |  |
| Disease location (rectum vs colon) | 1.708 (1.017 to 2.869) | **0.043** |  |  |  |  | 4.623 (1.704 to 12.543) | **0.003** |  | 5.011 (1.800 to 13.948) | **0.002** |
| TNM stage (I&II vs III) | 1.461 (0.863 to 2.474) | 0.158 |  |  |  |  | 0.309 (0.071 to 1.350) | 0.119 |  |  |  |
| Differentiation grade (well&moderately vs poorly) | 4.390 (1.585 to 12.158) | **0.004** |  | 4.757 (1.707 to 13.258) | **0.003** |  | 11.092 (2.512 to 48.985) | **0.001** |  | 19.722 (4.008 to 97.051) | **0.001*** |
| Chemotherapy (yes vs no) | 1.657 (0.908 to 3.026) | 0.100 |  |  |  |  | 0.986 (0.363 to 2.678) | 0.978 |  |  |  |
| Number of metastatic lymph nodes (≤ 3 vs > 3) | 2.138 (1.080 to 4.234) | **0.029** |  |  |  |  | 0.705 (0.093 to 5.337) | 0.735 |  |  |  |
| Serum CEA (ng/ml) (< 5 vs ≥ 5) | 1.447 (0.860 to 2.435) | 0.164 |  |  |  |  | 1.257 (0.456 to 3.463) | 0.658 |  |  |  |
| Serum CA19-9 (U/ml) (< 37 vs ≥ 37) | 1.667 (0.864 to 3.215) | 0.127 |  |  |  |  | 1.110 (0.253 to 4.862) | 0.890 |  |  |  |
| Abbreviations: HR = hazard ratio; TNM, tumor to node to metastasis; CEA, carcinoembryonic antigen; CA, carbohydrate antigen. *: P value < 0.001. | | | | | | | | | | | |

### Supplementary Table 11. Cox regression analysis of TAM abundances versus clinicopathological covariates with disease-free survival and overall survival in the validation cohort.

| Supplementary Table 11. Cox regression analysis of TAM abundances versus clinicopathological covariates with disease-free survival and overall survival in the validation cohort. | | | | | | | | | | | |
| --- | --- | --- | --- | --- | --- | --- | --- | --- | --- | --- | --- |
|  | Disease-free survival | | | | |  | Overall survival | | | | |
|  | Univariate analysis | |  | Multivariate analysis | |  | Univariate analysis | |  | Multivariate analysis | |
| Variables | HR (95% CI) | P-value |  | HR (95% CI) | P-value |  | HR (95% CI) | P-value |  | HR (95% CI) | P-value |
| TAMs (Low vs High) | 4.476 (2.472 to 8.104) | **0.001*** |  | 4.546 (2.495 to 8.282) | **0.001*** |  | 11.428 (3.273 to 39.903) | **0.001*** |  | 10.761 (3.066 to 37.772) | **0.001*** |
| Age (≤ 60 vs >60 years) | 1.310 (0.774 to 2.218) | 0.314 |  |  |  |  | 0.457 (0.169 to 1.235) | 0.123 |  |  |  |
| Gender (female vs male) | 0.564 (0.335 to 0.949) | **0.031** |  | 0.494 (0.291 to 0.837) | **0.009** |  | 0.507 (0.193 to 1.334) | 0.169 |  |  |  |
| Disease location (rectum vs colon) | 1.708 (1.017 to 2.869) | **0.043** |  |  |  |  | 4.623 (1.704 to 12.543) | **0.003** |  | 4.606 (1.647 to 12.879) | **0.004** |
| TNM stage (I&II vs III) | 1.461 (0.863 to 2.474) | 0.158 |  |  |  |  | 0.309 (0.071 to 1.350) | 0.119 |  |  |  |
| Differentiation grade (well&moderately vs poorly) | 4.390 (1.585 to 12.158) | **0.004** |  | 3.164 (1.136 to 8.813) | **0.028** |  | 11.092 (2.512 to 48.985) | **0.001** |  | 12.467 (2.637 to 58.930) | **0.001** |
| Chemotherapy (yes vs no) | 1.657 (0.908 to 3.026) | 0.100 |  |  |  |  | 0.986 (0.363 to 2.678) | 0.978 |  |  |  |
| Number of metastatic lymph nodes (≤ 3 vs > 3) | 2.138 (1.080 to 4.234) | **0.029** |  |  |  |  | 0.705 (0.093 to 5.337) | 0.735 |  |  |  |
| Serum CEA (ng/ml) (< 5 vs ≥ 5) | 1.447 (0.860 to 2.435) | 0.164 |  |  |  |  | 1.257 (0.456 to 3.463) | 0.658 |  |  |  |
| Serum CA19-9 (U/ml) (< 37 vs ≥ 37) | 1.667 (0.864 to 3.215) | 0.127 |  |  |  |  | 1.110 (0.253 to 4.862) | 0.890 |  |  |  |
| Abbreviations: HR = hazard ratio; TNM, tumor to node to metastasis; CEA, carcinoembryonic antigen; CA, carbohydrate antigen. *: P value < 0.001. | | | | | | | | | | | |
